# Supplementary material for: Deworming and micronutrient status by community open defecation prevalence: An observational study using nationally representative data from India, 2016–2018
Source: PLoS Med. 2024 May 10;21(5):e1004402. doi: 10.1371/journal.pmed.1004402 (PMC11125536; doi:10.1371/journal.pmed.1004402)
Supplement: S2 Fig — (DOCX) [file pmed.1004402.s003.docx]

**S2 Figure. Consumption of deworming medication in the previous six months among Indians aged 1-19 years by community open defecation level.**

**
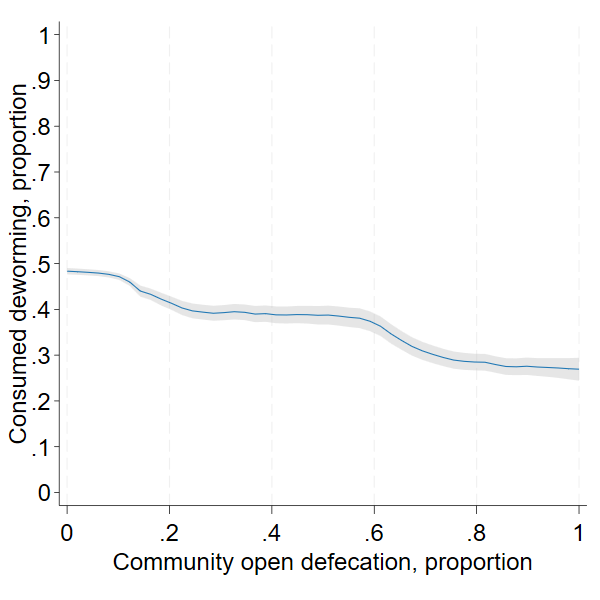
**
