# Supplementary material for: Deworming and micronutrient status by community open defecation prevalence: An observational study using nationally representative data from India, 2016–2018
Source: PLoS Med. 2024 May 10;21(5):e1004402. doi: 10.1371/journal.pmed.1004402 (PMC11125536; doi:10.1371/journal.pmed.1004402)
Supplement: S3 Fig — (DOCX) [file pmed.1004402.s004.docx]

**S3 Figure. Coefficients from regression of biomarkers on deworming status by community open defecation level among Indians aged 1-19 years.**


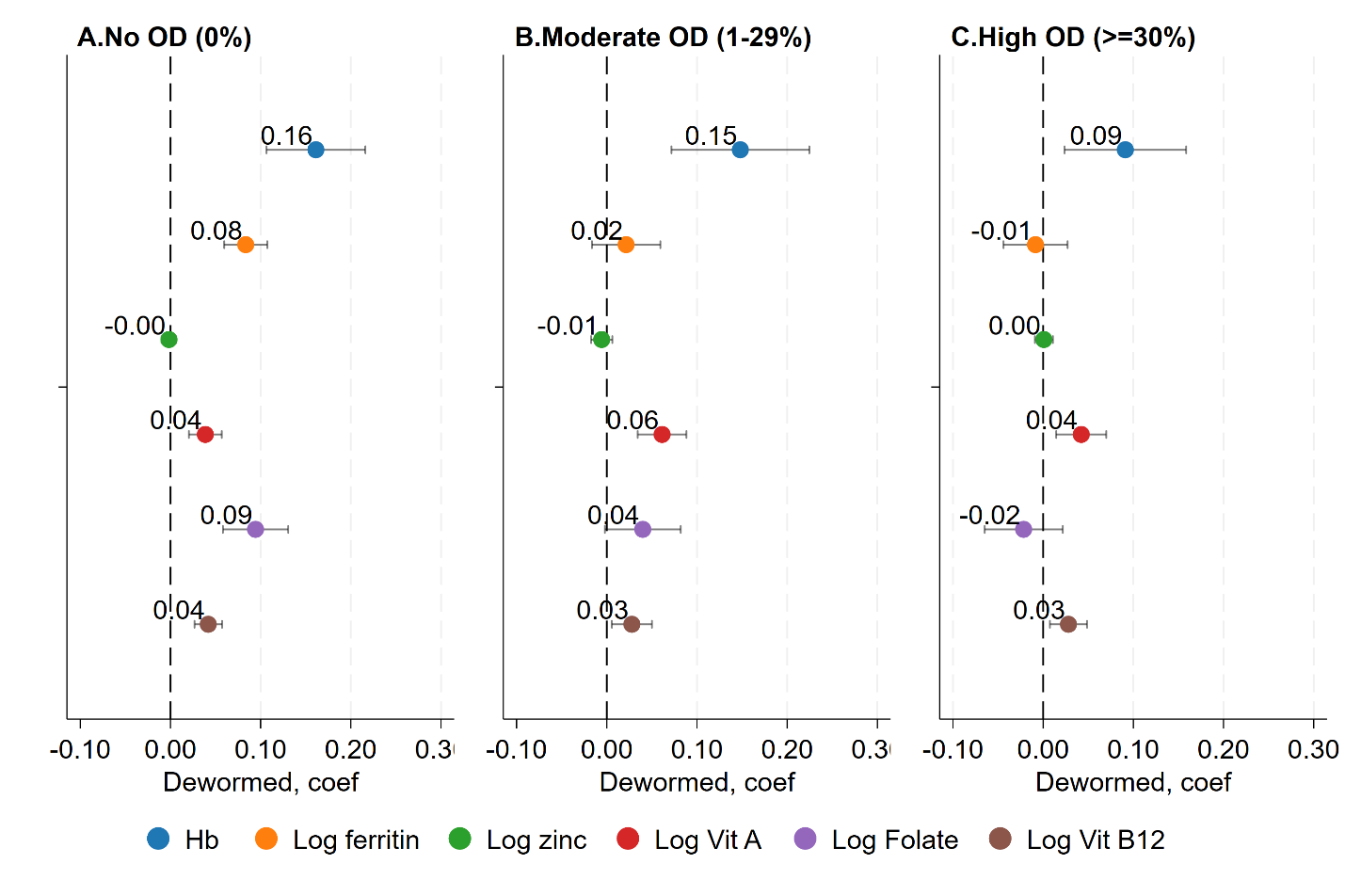


Regression models are adjusted for sex, age, parental education, wealth, dietary patterns, and seasonality. Standard error estimates are clustered at the PSU level. Open defecation (OD) was measured as the average proportion of sampled households practicing open defecation in a community, then was divided in 3 categories: OD free, moderate open defecation (1-29% of households in a community practicing OD), high open defecation (30-100% of households in a community practicing OD).
