# Supplementary material for: Deworming and micronutrient status by community open defecation prevalence: An observational study using nationally representative data from India, 2016–2018
Source: PLoS Med. 2024 May 10;21(5):e1004402. doi: 10.1371/journal.pmed.1004402 (PMC11125536; doi:10.1371/journal.pmed.1004402)
Supplement: S1 Table — (DOCX) [file pmed.1004402.s005.docx]

**S1 Table. Biomarker measurement and deficiency thresholds for outcomes**

| **Micronutrient/biomarker** | **Measurement method** | **Thresholds for deficiency or condition** | **Source** |
| --- | --- | --- | --- |
| Hemoglobin | Venous whole blood, using the cyanmethaemoglobin method | hb< 11.0 g/dl for children<5 yr, hb< 11.5 g/dl for children 5-11, hb< 12.0 g/dl for children 12-14 yr, hb< 12.0 g/dl for females >14yr, hb< 13.0 g/dl for males >14yr | World Health Organization |
| Serum ferritin (Iron) | Competitive immunoassay using direct  chemiluminescence (Siemens Centaur) | Serum ferritin level <15 μg/l | World Health Organization |
| Serum zinc | Atomic absorption spectrometry with D2 correction | Serum zinc <70 μg/dl for non-pregnant girls (morning fasting), <66 μg/dl for non-pregnant girls (morning non-fasting), <74 μg/dl for boys (morning fasting), <70 μg/dl for boys (morning non-fasting) | International Zinc Nutrition Consultative Group |
| Serum retinol (Vitamin A) | Concentration  in blood using HPLC reversed-phase chromatography | Serum retinol <20 μg/dL | World Health Organization |
| Erythrocyte folate | Competitive immunoassay using direct  chemiluminescence (Siemens Centaur) | Erythrocyte folate < 151 ng/ml | World Health Organization |
| Serum B12 | Competitive immunoassay using direct  chemiluminescence (Siemens Centaur) | Serum B12< 203 pg/ml | World Health Organization |
