# Supplementary material for: Deworming and micronutrient status by community open defecation prevalence: An observational study using nationally representative data from India, 2016–2018
Source: PLoS Med. 2024 May 10;21(5):e1004402. doi: 10.1371/journal.pmed.1004402 (PMC11125536; doi:10.1371/journal.pmed.1004402)
Supplement: S2 Table — (DOCX) [file pmed.1004402.s006.docx]

**S2 Table: Prevalence of anemia and micronutrient deficiencies among Indians aged 1-19 years by open defecation** **level.**

|  | **No open defecation** | | **Moderate open defecation** | | **High open defecation** | |
| --- | --- | --- | --- | --- | --- | --- |
|  | **%** | **95% CI** | **%** | **95% CI** | **%** | **95% CI** |
| Anemia | 21.5 | [20.9,22.0] | 26.9 | [26.0,27.7] | 31.0 | [30.2,31.9] |
| Iron deficiency | 24.7 | [24.1,25.4] | 28.0 | [27.1,28.9] | 23.0 | [22.1,23.8] |
| Zinc deficiency | 33.0 | [32.3,33.7] | 34.4 | [33.4,35.4] | 33.8 | [32.8,34.8] |
| Vitamin A deficiency | 15.0 | [14.5,15.6] | 15.6 | [14.8,16.3] | 20.2 | [19.3,21.1] |
| Folate deficiency | 28.8 | [28.2,29.4] | 32.1 | [31.2,33.0] | 36.3 | [35.4,37.3] |
| Vitamin B12 deficiency | 12.5 | [12.0,13.0] | 16.9 | [16.1,17.6] | 21.4 | [20.5,22.2] |

Open defecation (OD) was measured as the average proportion of sampled households practicing open defecation in a community, then was divided in 3 categories: OD free, moderate open defecation (1-29% of households in a community practicing OD), high open defecation (30-100% of households in a community practicing OD).
