# Supplementary material for: Deworming and micronutrient status by community open defecation prevalence: An observational study using nationally representative data from India, 2016–2018
Source: PLoS Med. 2024 May 10;21(5):e1004402. doi: 10.1371/journal.pmed.1004402 (PMC11125536; doi:10.1371/journal.pmed.1004402)
Supplement: S3 Table — (DOCX) [file pmed.1004402.s007.docx]

**S3 Table: Association between deworming, community OD, and their interaction with anemia and micronutrient deficiencies in India children.**

|  | **Anemia** | **Iron deficiency** | **Zinc deficiency** | **Vitamin A deficiency** | **Folate deficiency** | **Vitamin B12 deficiency** |
| --- | --- | --- | --- | --- | --- | --- |
|  | Odds ratio, [95%CI], (p-value) | | | | | |
| Dewormed, binary | 0.717 | 0.779 | 1.024 | 0.935 | 0.687 | 0.908 |
|  | [0.67,0.77] | [0.74,0.82] | [0.96,1.09] | [0.86,1.02] | [0.64,0.74] | [0.83,0.99] |
|  | (0.000) | (0.000) | (0.476) | (0.131) | (0.000) | (0.032) |
|  |  |  |  |  |  |  |
| Cluster OD, proportion | 1.247 | 0.809 | 0.838 | 1.551 | 0.845 | 1.895 |
|  | [1.06,1.46] | [0.69,0.95] | [0.67,1.04] | [1.20,2.00] | [0.66,1.09] | [1.54,2.34] |
|  | (0.007) | (0.010) | (0.113) | (0.001) | (0.190) | (0.000) |
|  |  |  |  |  |  |  |
| Dewormed x Cluster OD | 1.393 | 1.422 | 1.026 | 1.283 | 1.919 | 1.086 |
|  | [1.16,1.68] | [1.18,1.71] | [0.85,1.24] | [1.03,1.60] | [1.59,2.32] | [0.89,1.32] |
|  | (0.000) | (0.000) | (0.797) | (0.028) | (0.000) | (0.411) |
|  |  |  |  |  |  |  |
| Observations | 41368 | 37035 | 33909 | 32214 | 40600 | 34014 |

Open defecation (OD) was measured as the average proportion of sampled households practicing open defecation in a community. Regression models are adjusted for sex, age, parental education, wealth, dietary patterns, and seasonality. Standard error estimates are clustered at the PSU level.
