# Supplementary material for: Deworming and micronutrient status by community open defecation prevalence: An observational study using nationally representative data from India, 2016–2018
Source: PLoS Med. 2024 May 10;21(5):e1004402. doi: 10.1371/journal.pmed.1004402 (PMC11125536; doi:10.1371/journal.pmed.1004402)
Supplement: Attachment — Submitted filename: chakrabarti.pdf [file pmed.1004402.s009.pdf]

This paper uses survey data to examine the association between open defecation (OD) , deworming and nutritional markers in children in India.

I was asked for a methodological report and I interpret that to include all aspects of the design and conduct of the study. The version I received did not have page numbers. My numbering has the abstract starting on page 1

# Points of detail

**Page 4** Is junk food a well–defined category of food?

**Page 4** Does that mean that December to February is treated as the reference category?

**Page 5** Since categorising an essentially continuous variable wastes information (Altman and Royston, 2006; Royston et al., 2006) and leads to models which are often implausible as they predict the effect remaining flat within categories and then jumping to a new value at the category boundary I wonder why the authors only present the model with continuous OD as a sensitivity analysis. To my mind it is clearly superior and has the additional benefit of detecting possible thresholds.

**Page 5, Table 1** I do not understand what the meaning of averaging the wealth categories is. Strictly speaking the (four) quintiles are values on the principal component but it looks as though this is just the mean of {1, 2, 3, 4, 5}

**Page 7 and Figure 2** If the authors are relying on the confidence intervals to support their statement about vitamin A then I think more care is needed. Just because the confidence intervals for for the three scenarios do not overlap does not mean that they are not different in the statistical significance sense of different (Sedgwick, 2012). Vitamin A is monotonic with OD exposure as well.

**Page 8** There seems to me to be more going on in Figure 3 than the text describes. The results for folate seem quite surprising as they indicate that in locations of high OD deworming is harmful.

**Page 8** I do not use Stata so citing the Margins command is not very helpful to m or to other non–Stata people. Is it possible to describe what it does in words? This is not the case for some of the others.

**Page 9** Does the phrase in moderate and high OD contexts' imply that there was an effect in the low OD context?

**Figure S1** This is rather more dramatic than it should be since the $y$–axis zero has been suppressed. It is still fairly dramatic though as it reveals a halving of deworming over the range of OD.

# Points of more substance

## Interaction

The authors have used separate regressions and have relied on eye–balling differences between them (pages 5, 7, and 9 are examples). I wonder why they have gone to such lengths to avoid fitting a model with interaction term(s). In fact the word interaction when it does occur in the text is used in other contexts. An interaction would enable us to see the change in (log) odds associated with a unit change in OD.

## Who uses deworming?

The quite strong relationship between OD and deworming seems to me quite important. This is shown in Figure S1. This implies to me that the children who receive deworming are different in different areas. There must be a variety of social, economic and cultural reasons why people defecate in the open which could, in principle, be associated with propensity to deworm your child. How does this affect the public health message which the authors wish us to draw?

# Summary

A few points for clarification and a couple of things which puzzle me.

Michael Dewey

# References

D G Altman and P Royston. The cost of dichotomising continuous variables. *British Medical Journal*, 332:1080, 2006.

P Royston, D G Altman, and W Sauerbrei. Dichotomizing continuous predictors in multiple regression: a bad idea. *Statistics in Medicine*, 25:127–141, 2006.

P Sedgwick. Confidence intervals and statistical significance. *British Medical Journal*, 344(e2238), 2012.
