# Supplementary material for: Deworming and micronutrient status by community open defecation prevalence: An observational study using nationally representative data from India, 2016–2018
Source: PLoS Med. 2024 May 10;21(5):e1004402. doi: 10.1371/journal.pmed.1004402 (PMC11125536; doi:10.1371/journal.pmed.1004402)
Supplement: Attachment — Submitted filename: Deworming_OD_Response to reviewers final_20032024.docx [file pmed.1004402.s010.docx]

**Reviewer 1**

**Comment 1:** I am grateful to have been afforded the opportunity to review this manuscript. Despite its potential importance to the readers, this manuscript requires revisions. Below are a few considerations the authors could take into account in order to fortify the manuscript.

**Response:** We thank the reviewer for offering suggestions to improve the manuscript.

**Comment 2:** Title: Enhancing the clarity and relevance of the title: The title should be direct, accurate, interesting, concise, precise and unique.

**Response:** We have revised the title as suggested and according to the journal style. The old title was “THE UTILITY OF DEWORMING TO ADDRESS ANEMIA AND MICRONUTRIENT DEFICIENCIES IN COMMUNITIES THAT PRACTICE OPEN DEFECATION: NATIONALLY REPRESENTATIVE EVIDENCE FROM INDIA” and the new title is “DEWORMING AND MICRONUTRIENT STATUS BY COMMUNITY OPEN DEFECATION PREVALENCE: A MODELLING STUDY FROM INDIA”.

**Comment 3:**

**Abstract:**
1. The abstract could be improved by condensing highlighting key content areas and eliminating irrelevant details.

**Response:** We have restructured the abstract per the journal’s guidelines.

**Comment 4:**

2.      Authors should consider incorporating a discussion to include implications for policy and public health interventions.

**Response:** We have amended the conclusion of the abstract as follows:

*Line 42: Public health policy could involve sequencing interventions, with focus on improving deworming coverage in communities that have achieved minimum thresholds of OD and re- triggering sanitation interventions in high OD communities prior to deworming days, ensuring high coverage for both. The efficacy of micronutrient supplementation as a complementary strategy to improve nutritional outcomes alongside deworming and OD elimination in this age group needs further study.*

**Comment 5:**

Introduction
1.      Line 57: The citation is missing, please cite

**Response:** We thank the reviewer for identifying this missing reference. We have added the missing reference.

*Guideline: preventive chemotherapy to control soil-transmitted helminths infections in at risk population groups. Geneva: World Health Organization; 2017. Licence: CC BY-NC-SA 3.0 IGO.*

**Comment 6:**

2.      It may be beneficial to provide more background information on the relation between STH exposure and anemia or micronutrient deficiencies to better understand the hypothesis being tested.

**Response:** We thank the reviewer for this suggestion. We have added the following lines in the introduction.

*Line 79: While Ascaris has been reported to be the most prevalent STH infection globally, hookworm infections are linked to the highest DALYs and leads to iron deficiency anemia, hypoalbuminemia, and hypoproteinemia due to chronic blood loss especially in susceptible populations with low iron reserves such as pregnant women (leading to intra-uterine growth retardation and low birth weights) and young children (leading to stunting and cognitive deficits) and in heavy intensity infections. Symptoms are also dependent on species with A. duodenale leading to 10 times as much blood loss as that caused by N. americanus. Ascaris leads to malnutrition, vitamin A and C deficiency, cognitive deficits and lack of physical fitness in children with high worm burdens due to anorexia and absorption defects. Due to large worm size this can also lead to acute intestinal and biliary tract obstruction. In trichuriasis, inflammation of large intestine and blood loss can lead to colitis (especially in heavy intensity infections), iron deficiency anemia and stunting as well as diarrhea in children.*

**Comment 7:**

Methods
1.      Line 117: The authors should clarify whether this study employed secondary data analysis

**Response:** We thank the reviewer for this comment. We have added the words “cross-sectional secondary” data in the first line.

*Line 131: We used cross-sectional secondary data from the Comprehensive National Nutrition Survey (CNNS) published by the Ministry of Health and Family Welfare (MoHFW), Government of India, UNICEF and the Population Council.*

**Comment 8:**

2.      The author should include more information on how the samples were handled and processed.

**Response:** We thank the reviewer for this comment. We have now added more information on how samples were handled and processed.

*Line 157: Blood was collected in the morning via antecubital venipuncture by 360 trained phlebotomists (qualified with a Diploma in Medical Laboratory Technology). Eight milliliters (ml) of blood were collected from children 1–4 years and 10 ml from children 5–19 years. Once collected, the vacutainer tubes with blood samples were placed in cool boxes without direct contact with the icepacks and transported to the nearest pre-identified collection center at appropriate temperatures. The blood samples for serum retinol were covered with aluminum foil soon after collection to protect against light exposure. At the collection center, the blood samples were spun for 20 minutes and aliquoted into appropriately sized tubes for laboratory testing and aliquots were stored for laboratory analysis (plasma at 5–7°C, serum frozen)*

**Comment 9:**

Results
1.      Table 1: Superscript 5 is missing in the note

**Response:** We thank the reviewer for identifying this omission. We have added the superscript.

**Comment 10:**

2.      Authors should provide an overview of the potential confounding variables that may have affected the outcome of their study

**Response:** We thank the reviewer for this comment. We have added the following line in the discussion section reflecting these limitations.

*Line 361: Biases in observational cross-sectional studies can arise due to unmeasured omitted confounders including variables that exhibit correlation with deworming, micronutrient status, and OD, that were not included in our study. For example, some communities may have poor governance leading to poor health and sanitation services.*

**Comment 11:**

Discussion
1.      The authors should consider expanding on the implications of their findings and discussing potential strategies to address deworming coverage and high OD prevalence

**Response:** We added the following sentences.

*Line 451: Interventions to reduce OD, and keep it low, might include community-led triggering and retriggering events and social marketing to support local latrine manufacture and construction (Chirgwin et al., 2021), and maintenance efforts to extend the lives of the pits - and therefore latrine use - such as the construction of double-pits where space permits, subsidies for pit emptying and vermifiltration (Sioné et al., 2023). Participation in deworming and therefore coverage of targeted populations is influenced by several factors such as gender, socioeconomic status, population density, school attendance, local religious and cultural beliefs, awareness of helminth infections and trust in public health campaigns. Community engagement and sensitization activities need to be tailored to address these factors in the form of media announcements, social media or local dissemination by trained and motivated community drug distributors (including schoolteachers) prior to national deworming campaigns.*

**Comment 12:**

General comment
The manuscript exhibits promising potential to captivate its readers. Nevertheless, it requires minor revisions for optimal refinement

**Response:** We thank the reviewer for this comment.

**Reviewer 2 (Michael Dewey)**

**Comment 1:** This paper uses survey data to examine the association between open defecation (OD), deworming and nutritional markers in children in India.

I was asked for a methodological report, and I interpret that to include all aspects of the design and conduct of the study. The version I received did not have page numbers. My numbering has the abstract starting on page 1

**Response:** We thank the reviewer for their methodological review.

**Comment 2:** Page 4 Is junk food a well-defined category of food?

**Response:** We thank the reviewer for this comment. In the questionnaire “junk food” was indicated by examples including burger, pizza, pasta, and instant noodles. We have added these and details of other food groups in the unhealthy category to the methods section.

*Line 186: To account for diet, a significant predictor of micronutrient status[39], we constructed indicators which represented the number of food groups (at least weekly consumption of foods in a category) in three categories - animal-sourced foods (milk or milk products, eggs, fish, chicken, and meat), plant-sourced foods (pulses or beans, green leafy vegetables, other vegetables, fruits, and nuts/seeds), and unhealthy foods (fried foods (poori, pakora, vada, samosa, tikki), junk food (burger, pizza, pasta, instant noodles), sweets (Indian sweets, pastries/cakes, donuts), and sugar sweetened beverages.*

**Comment 3:** Page 4 Does that mean that December to February is treated as the reference category?

**Response:** Yes. December to February was treated as the reference. We have added a line to the methods to reflect this.

*Line 200: December to February was treated as the reference category.*

**Comment 4:** Page 5 Since categorizing an essentially continuous variable wastes information (Altman and Royston, 2006; Royston et al., 2006) and leads to models which are often implausible as they predict the effect remaining at within categories and then jumping to a new value at the category boundary, I wonder why the authors only present the model with continuous OD as a sensitivity analysis. To my mind it is clearly superior and has the additional benefit of detecting possible thresholds.

D G Altman and P Royston. The cost of dichotomizing continuous variables.

British Medical Journal, 332:1080, 2006.

P Royston, D G Altman, and W Sauerbrei. Dichotomizing continuous predictors in multiple regression: a bad idea. Statistics in Medicine, 25:127{141,

2006.

**Response:** We thank the reviewer for this comment. Our motivation behind using categories for OD was to simplify the analysis for a broader audience or readers who may have difficulties interpreting results from models with interaction terms.

Per recommendations of the reviewer, we have moved the models with categories to sensitivity analyses.

Further, responding also to comment 11, we agree with the reviewer that an interaction model is most appropriate, so that is now our primary specification. The model presented in previous Figure 3 (now Figure 2) uses an interaction term to fit predicted margins. We have described the model in the methods section in greater detail and reported the results from this model as our main results. We have also added Table S3 to the supplementary material that reports the coefficients from the interaction model.

*Methods*

*Line 206: Thirdly, we used logistic regression to (1) examine the association between deworming and anemia or micronutrient deficiency outcomes, and (2) identify a minimum OD rate after which deworming becomes ineffective. Explanatory variables included in the model were deworming (binary), cluster OD (continuous ranging from 0, zero OD prevalence to 1, maximum OD prevalence) and their interaction. All other covariates were also included in these models* *to reduce confounding. All regressions were clustered at the PSU-level to account for correlation of outcomes within communities. The interaction term models that the association of deworming with the outcome depends on the level of OD. A positive and significant interaction term implies that the benefits of deworming for the given outcome are diminished in communities with OD.*

*We performed two sets of sensitivity analyses. Firstly, to fit a “within sanitation context” model, separate regressions were run for each outcome for the three OD sub-samples (OD free, moderate OD, and high OD) to gauge changes in regression coefficients by varying OD context.*

*Results*

*Line 268: Anemia, vitamin A, folate, and vitamin B12 deficiencies were greatest in communities with high OD, and higher in communities with moderate OD compared to OD-free communities (Table S2). Table S3 shows results from the fully adjusted logit regression model examining interactions between deworming and cluster OD. In communities with no OD (cluster OD=0), dewormed children had significantly lower odds of being anemic Odds ratio (OR)=0.71; (95%CI [0.66,0.76],p<0.001), iron deficient OR=0.78; (95%CI [0.74,0.82], p<0.001), folate deficient OR=0.69; (95%CI [0.64,0.74], p<0.001) and vitamin B12 deficient OR=0.91; (95%CI [0.83,0.99], p<0.001), relative to non-dewormed children. Among non-dewormed children (dewormed=0), maximum cluster OD (cluster OD=1) was associated with significantly higher odds of anemia OR=1.24; (95%CI [1.06,1.46], p=0.003), vitamin A deficiency OR=1.55; (95%CI [1.20,2.00], p<0.001) and Vitamin B12 deficiency OR=1.89; (95%CI [1.54,2.34], p<0.001). Cluster OD significantly modified the association between deworming and micronutrient deficiencies, revealed by significant coefficients on interaction terms for anemia, iron, vitamin A, and folate deficiencies. The predicted marginal effects from these interactions are shown in Figure 2, revealing that, across outcomes, deworming begins to become ineffective when approximately 30-60% of households in a community practice OD. Beyond this level, the confidence intervals of the predicted margins among dewormed individuals overlap with non-dewormed individuals for anemia, iron, and folate deficiency. Moreover, folate deficiencies were higher among dewormed children, but the predicted margins had very high uncertainty at high OD levels.*

**Comment 5:** Page 5, Table 1 I do not understand what the meaning of averaging the wealth categories is. Strictly speaking the (four) quintiles are values on the principal component but it looks as though this is just the mean of f1, 2, 3, 4, 5g

**Response:** Yes, the reviewer is correct, this was just the average of the 5 quintiles. For clarity we have now shown the percentages of the wealth quintiles in the revised version of Table 1.

**Comment 6:** Page 7 and Figure 2 If the authors are relying on the confidence intervals to support their statement about vitamin A then I think more care is needed. Just because the confidence intervals for the three scenarios do not overlap does not mean that they are not different in the statistical significance sense of different (Sedgwick, 2012). Vitamin A is monotonic with OD exposure as well.

P Sedgwick. Confidence intervals and statistical significance. British Medical

Journal, 344(e2238), 2012.

**Response:** We thank the reviewer for this comment. We have amended statements for Vitamin-A to clarify the reviewer’s concern.

*Line 303: An attenuation of dose response was also observed for iron, folate deficiency, and Vitamin A, moving the odds ratio on deworming closer to the null with increasing OD, where the difference in deficiency following deworming in no OD communities versus high OD communities was statistically significant for iron and folate. In no OD communities, Vitamin A deficiencies were lower among dewormed children OR=0.92; (95%CI [0.83,1.01], p=0.100).*

**Comment 7:** Page 8 There seems to me to be more going on in Figure 3 than the text describes. The results for folate seem quite surprising as they indicate that in locations of high OD deworming is harmful.

**Response:** We thank the reviewer for this comment. We have amended our statement for this observation in the results section.

*Line 282: Beyond this level, the confidence intervals of the predicted margins among dewormed individuals overlap with non-dewormed individuals for anemia, iron, and folate deficiency. Moreover, folate deficiencies were higher among dewormed children, but the predicted margins had high uncertainty at higher OD levels.*

**Comment 8:** Page 8 I do not use Stata so citing the Margins command is not very helpful to me or to other non-Stata people. Is it possible to describe what it does in words? This is not the case for some of the others.

**Response:** We have added the following sentences in the methods section to address this concern.

*Line 214: For plotting these complex relationships, we used the Stata ‘margins’ package. Margins calculates the predicted level of the outcome for specific points through the exposure distribution (OD) separately for dewormed and non-dewormed children, holding all other variables in the model constant. We predicted marginal effects at 10% increments from 0 to 100% OD.*

**Comment 9:** Page 9 Does the phrase in moderate and high OD contexts' imply that there was an effect in the low OD context?

**Response:** The statement implies that there was a statistically significant association in the no OD sub-sample for log ferritin, log folate and log vitamin B12.

**Comment 10:** Figure S1 This is rather more dramatic than it should be since the yaxis zero has been suppressed. It is still fairly dramatic though as it reveals a halving of deworming over the range of OD.

**Response:** We have revised the figure by extending the y-axis from 0 to 100%. The revised figure is below.

**Comment 11:** The authors have used separate regressions and have relied on eye balling differences between them (pages 5, 7, and 9 are examples). I wonder why they have gone to such lengths to avoid fitting a model with interaction term(s). In fact, the word interaction when it does occur in the text is used in other contexts. An interaction would enable us to see the change in (log) odds associated with a unit change in OD.

**Response:** Please see response to comment 4.

**Comment 12:** The quite strong relationship between OD and deworming seems to me quite important. This is shown in Figure S1. This implies to me that the children who receive deworming are different in different areas. There must be a variety of social, economic and cultural reasons why people defecate in the open which could, in principle, be associated with propensity to deworm your child. How does this affect the public health message which the authors wish us to draw?

**Response:** We thank the reviewer for this suggestion. We have added the following lines in the discussion to address this.

*Line 406: Moreover, consumption of deworming medication is lower, on average, in communities where the prevalence of OD is high. This inverse relationship between OD and deworming levels is likely attributable to weaker public health services in such communities, suggesting a need to identify such hotspots and prioritize improvements in sanitation services, messaging to reduce OD, and the distribution of deworming pills at schools, Anganwadi centers, and by frontline health workers in such areas.*

**Reviewer 3**

**Comment 1:** I would like to congratulate the authors on this valuable paper. This provides comprehensive information from a very big national survey on the range of nutrition interactions between open defecation, deworming and infections. This is crucial for policy making around WASH interventions. I have a few very minor comments here. But, overall the paper is very good.

**Response:** We thank the reviewer for their kind appreciation of the manuscript.

**Comment 2:** Line 57 - There is a marker for a reference that you have forgotten to put in.

**Response:** We thank the reviewer for identifying this missing reference. We have added the missing reference.

*Guideline: preventive chemotherapy to control soil-transmitted helminths infections in at risk population groups. Geneva: World Health Organization; 2017. Licence: CC BY-NC-SA 3.0 IGO.*

**Comment 3:** Line 64 - I don't understand the logic of this last sentence. I think there is the need to consider sanitation interventions ,as well as behaviour change communication as your results show.

**Response:** We have amended the sentence as follows:

*Line 64: Therefore, it may be important to consider the relative roles and timing of deworming alongside efforts to improve sanitation access at the household level (the necessary condition to reduce open defecation) and behavior change communication activities promoting sanitary practices (the sufficient condition), when designing public health programs that aim to reduce morbidity and worm burden in particular contexts.*

**Comment 4:** Line 78-80 - Please include a reference for the first two sentences here regarding the numerous factors contributing to STH in India.

**Response:** We have added the reference.

*Epidemiology of soil transmitted helminths and risk analysis of hookworm infections in the community: Results from the deworm3 trial in southern india. Ajjampur S, Kaliappan S, […] Walson J. PLoS Neglected Tropical Diseases (2021) 15(4)*

**Comment 5:** Line 108 - I think this sentence would be more effective if you included the direction of the association between deworming and outcomes. For example: we hypothesise the association between deworming and reductions in anemia or micronutrient deficiencies....

**Response:** We thank the reviewer for this suggestion. We have revised the line as follows.

*Line 122: In this study, we hypothesize that the negative association between deworming and anemia or micronutrient deficiencies will move closer to the null with increasing community OD levels, due to significant STH exposure and re- infections in such areas.*

**Comment 6:** Line 125 - survey conducted in 30 out of how many states in India? will give us a better picture of the scale of the survey.

**Response:** We have amended the lines to reflect the total number of states and union territories in India.

*Line 147: Our analytical sample comprised 105,060 children aged 1-19 years from 30 out of 37 Indian states and union territories and 2035 communities (PSUs).*

**Comment 7:** Line 157 - You could describe in a bit more detail how the nutrition information was collected. ie food frequency questionnaire? using a validated instrument?

**Response:** We have added the following details about the instrument used to collect diet data.

*Line 185: CNNS used a pre-validated short food frequency questionnaire to collect data on the number of days per week individuals consumed any amounts of 17 food groups.*

**Comment 8:** Line 157 - I don't understand the dietary diversity variable. You have not used a standard procedure for assessing dietary diversity, and it seems you include 'unhealthy' foods as one of the components of dietary diversity. Could you explain a bit more why you use this approach? And in the Results you don't actually seem to use this dieatry diversity measure, you just report separately for each food type.

**Response:** We thank the reviewer for pointing this out. We agree that the description of the diet variables in the methods was confusing. We have revised it as follows.

*Line 186: To account for diet, a significant predictor of micronutrient status, we constructed indicators which represented the number of food groups (at least weekly consumption of foods in a category) in three categories - animal-sourced foods (milk or milk products, eggs, fish, chicken, and meat), plant-sourced foods (pulses or beans, green leafy vegetables, other vegetables, fruits, and nuts/seeds), and unhealthy foods (fried foods (poori, pakora, vada, samosa, tikki), junk food (burger, pizza, pasta, instant noodles), sweets (Indian sweets, pastries/cakes, donuts), and sugar sweetened beverages. Within each of the three categories, we counted the number of food groups that were consumed in the previous week to proxy the intake of healthy and unhealthy foods. Given India’s large vegetarian population, we split the healthy foods into two indicators, plant, and animal-based foods. A lack of data on some food groups including dark leafy greens and vegetables, or Vitamin-A rich fruits and vegetables, precluded calculation of the standard dietary diversity indicator.*

**Comment 9:** Finally Table 2 is missing from the file i received. I could basically understand from the Results text what was there but i need to see the Table as well.

**Response:** We thank the reviewer for pointing this out. We have added Table 2.

**Reviewer 4**

**Comment 1:** Chakrabarti and colleagues present an interesting secondary analysis of India's national nutrition survey to investigate the relationships between open defecation (OD), deworming, and nutritional status. The analyses show that the impact of deworming on micronutrient status is modified by the level of OD in the community. The analysis is appropriate for the data and the interpretation is sound. However, there are several issues that would benefit from careful consideration, including the following issues.

**Response:** We thank the reviewer for their kind appreciation of the manuscript.

**Comment 2:** Numerous studies have reported associations between the risk of STH and access to WASH. However, cross-sectional studies are subject to systematic errors or biases, and the association between WASH and STH is not consistently supported by the available evidence from randomized controlled trials. These trials report varied effects of WASH interventions on infection risk. The potential reasons for the incongruence between cross-sectional studies and trials are discussed in more detail in the following viewpoint: <https://eur03.safelinks.protection.outlook.com/?url=https%3A%2F%2Fparasitesandvectors.biomedcentral.com%2Farticles%2F10.1186%2Fs13071-019-3532-6&data=05%7C02%7Csamuel.scott%40cgiar.org%7C9bca2e95c0354359ba1808dc389bf0a5%7C6afa0e00fa1440b78a2e22a7f8c357d5%7C0%7C0%7C638447489870130370%7CUnknown%7CTWFpbGZsb3d8eyJWIjoiMC4wLjAwMDAiLCJQIjoiV2luMzIiLCJBTiI6Ik1haWwiLCJXVCI6Mn0%3D%7C0%7C%7C%7C&sdata=LCzxc7nQyyX9quaZroBkv9n%2FQX0KgNlHX%2FOUk5eAvno%3D&reserved=0>

**Response:** We appreciate the reviewer's input on the WASH randomized controlled trials. Many WASH researchers, including us, see them as highlighting the necessity for holistic interventions. However, to address the reviewers point, we have added the following lines to the discussion section.

*Line 378: Cross-sectional studies are subject to systematic errors or biases, and the association between WASH and STH is not consistently supported by the available evidence from randomized controlled trials. Conventional approaches often focus on low-cost household interventions, neglecting broader fecal exposure risks linked to undernutrition. Moreover, to estimate the causal interaction effects of deworming on micronutrient status in different community sanitation contexts would require researchers to randomize deworming treatments and sanitation levels across communities. In practical terms, such a study would be very difficult to implement. Thus, it is prudent to rely on large scale observational datasets to inform programs and policies until such trials are conducted.*

**Comment 3:** The relationship between STH, micronutrient status and OD is likely to differ depending on the specific STH species, owing to differences in life cycle and morbidity. While the available data do not permit a species-level analysis, it is essential for the discussion to address this aspect. This should include a discussion of the geographical distribution of each species in India, as the results might vary by geography.

**Response:** We thank the reviewer for this suggestion. We have added lines in the introduction about STH related morbidity:

*Line 79: While Ascaris has been reported to be the most prevalent STH infection globally, hookworm infections are linked to the highest DALYs and leads to iron deficiency anemia, hypoalbuminaemia and hypoproteinaemia due to chronic blood loss especially in susceptible populations with low iron reserves such as pregnant women (leading to intra-uterine growth retardation and low birth weights) and young children (leading to stunting and cognitive deficits) and in heavy intensity infections. Symptoms are also dependent on species with A. duodenale leading to 10 times as much blood loss as that caused by N. americanus. Ascaris leads to malnutrition, vitamin A and C deficiency, cognitive deficits and lack of physical fitness in children with high worm burdens due to anorexia and absorption defects. Due to large worm size this can also lead to acute intestinal and biliary tract obstruction. In trichuriasis, inflammation of large intestine and blood loss can lead to colitis (especially in heavy intensity infections), iron deficiency anemia and stunting as well as diarrhea in children.*

We have also added lines in the discussion about worm species in India:

*Line 424: STH prevalence is influenced by climatic conditions (especially land surface temperature, humidity) and soil structure that favor embryonation of ova (Ascaris and Trichuris) and larval development and survival (hookworms). STH exhibit considerable geographic heterogeneity in distribution in India, both due to these climatic and soil differences and due to socio-demographic, migration and hygiene behavioral patterns as well as previous rounds of deworming. STH are also known to vary at finer spatial scales of village or community within districts and have been linked to poor household WASH indicators in nearly all surveys. Surveys from India have indicated higher levels of Ascaris in north and northeastern India and in urban populations and higher levels of hookworm infections in southern India and in rural populations in some studies, although the reliability of these wide ranging estimates are affected by the populations surveyed (age groups included, sampling at health centers or schools) and quality of stool screening carried out (methodology used and whether screened rapidly enough to ensure detection of hookworm ova).*

**Comment 4:** Although the results are intuitive, their accuracy relies on the potential for reporting bias in deworming and OD. This aspect requires thorough discussion.

**Response:** We have added the following line in the discussion section reflecting these limitations.

*Line 364: Secondly, deworming status was self-reported by respondents and OD was estimated from observations on a sample of households from the community thereby creating the potential for measurement error which may bias our estimates towards the null.*

**Comment 5:** The cross-sectional design of the analysis introduces the possibility of unobserved confounding, and it would be important to consider potential confounders in the discussion.

**Response:** We have added the following line in the discussion section reflecting these limitations.

*Line 361: Biases in observational cross-sectional studies can arise due to unmeasured omitted confounders including variables that exhibit correlation with deworming, micronutrient status, and OD, that were not included in our study. For example, some communities may have poor governance leading to poor health and sanitation services.*

**Comment 6:** This pertains to the style of the journal, but many results are presented both within the text and in tables, and there is potential to minimize redundancies in reporting.

**Response:** We have followed the journal guidelines for reporting results in the revised version.

**Comment 7:** It would be useful to specify the coefficients of the polynomial smoothing used to develop age profiles of deworming and OD.

**Response:** Stata doesn’t provide coefficients for polynomial smoothing, so we are unable to report coefficients. However, to provide more detail, we have specified the following in the methods section.

*Line 205: We used STATA defaults for plotting the curves with the epanechnikov kernel, a rule-of-thumb bandwidth, and a zero-degree polynomial (local-mean smoothing).*

**Editor**

**Comment 1:** Please revise your title according to PLOS Medicine's style. Your title must be nondeclarative and not a question. It should begin with main concept if possible. "Effect of" should be used only if causality can be inferred, i.e., for an RCT. Please place the study design ("A randomized controlled trial," "A retrospective study," "A modelling study," etc.) in the subtitle (ie, after a colon).

**Response:** We have revised the title according to PLOS Medicine’s style. The old title was “THE UTILITY OF DEWORMING TO ADDRESS ANEMIA AND MICRONUTRIENT DEFICIENCIES IN COMMUNITIES THAT PRACTICE OPEN DEFECATION: NATIONALLY REPRESENTATIVE EVIDENCE FROM INDIA” and the new title is “DEWORMING AND MICRONUTRIENT STATUS BY COMMUNITY OPEN DEFECATION PREVALENCE: A MODELLING STUDY FROM INDIA”.

**Comment 2:** Please structure your abstract using the PLOS Medicine headings (Background, Methods and Findings, Conclusions). In the last sentence of the Abstract Methods and Findings section, please describe the main limitation(s) of the study's methodology.

**Response:** We have restructured the abstract per the journal’s guidelines. We have also added the main limitation to the methods section of the abstract.

**Comment 3:** Please ensure that the study is reported according to the STROBE guideline, and include the completed STROBE checklist as Supporting Information.  Please add the following statement, or similar, to the Methods: ""This study is reported as per the Strengthening the Reporting of Observational Studies in Epidemiology (STROBE) guideline (S1 Checklist).The STROBE guideline can be found here: <https://eur03.safelinks.protection.outlook.com/?url=http%3A%2F%2Fwww.equator-network.org%2Freporting-guidelines%2Fstrobe%2F&data=05%7C02%7Csamuel.scott%40cgiar.org%7C9bca2e95c0354359ba1808dc389bf0a5%7C6afa0e00fa1440b78a2e22a7f8c357d5%7C0%7C0%7C638447489870139051%7CUnknown%7CTWFpbGZsb3d8eyJWIjoiMC4wLjAwMDAiLCJQIjoiV2luMzIiLCJBTiI6Ik1haWwiLCJXVCI6Mn0%3D%7C0%7C%7C%7C&sdata=LZZvG9d1r4vCpGrpJ%2FY3ff0ONy%2FMPtNc%2FclU5azvUtY%3D&reserved=0>
When completing the checklist, please use section and paragraph numbers, rather than page numbers."

**Response:** We have completed the checklist.

**Comment 4:** Discussion: please present and organize the Discussion as follows: a short, clear summary of the article's findings; what the study adds to existing research and where and why the results may differ from previous research; strengths and limitations of the study; implications and next steps for research, clinical practice and/or public policy implications; followed by a one-paragraph conclusion. Please remove all subheadings within your Discussion.

**Response:** We have organized the discussion per the instructions.

**Comment 5:** References: Please use the "Vancouver" style for reference formatting, and see our website for other reference guidelines <https://eur03.safelinks.protection.outlook.com/?url=https%3A%2F%2Fjournals.plos.org%2Fplosmedicine%2Fs%2Fsubmission-guidelines%23loc-references&data=05%7C02%7Csamuel.scott%40cgiar.org%7C9bca2e95c0354359ba1808dc389bf0a5%7C6afa0e00fa1440b78a2e22a7f8c357d5%7C0%7C0%7C638447489870143556%7CUnknown%7CTWFpbGZsb3d8eyJWIjoiMC4wLjAwMDAiLCJQIjoiV2luMzIiLCJBTiI6Ik1haWwiLCJXVCI6Mn0%3D%7C0%7C%7C%7C&sdata=kVg4PmktswVRpmluZp4PYJTpybSW7TD3ZL0jBgj5YsI%3D&reserved=0>.

**Response:** We have made this change.

**Comment 6:** For in-text reference, citations are placed within square parentheses and should precede punctuation. Please amend throughout.

**Response:** We have made this change.

**Comment 7:** Where you provide CI values, please also provide p values for all results where appropriate (including the abstract), check and amend throughout. We suggest reporting statistical information in the following format: ‘x’; (95% CI [‘y’,’ z’] p value). We suggest use of commas as opposed to hyphens (as these can be confused with negative values) to separate upper and lower bounds. For p values, please report as p<0.001 and where higher as 'p=0.002'. Please add the statistical method used to your method section.

**Response:** We have made this change.

**Comment 8:** In addition, we request that you upload any figures associated with your paper as individual TIF or EPS files with 300dpi resolution at resubmission; please read our figure guidelines for more information on our requirements: <https://eur03.safelinks.protection.outlook.com/?url=http%3A%2F%2Fjournals.plos.org%2Fplosmedicine%2Fs%2Ffigures&data=05%7C02%7Csamuel.scott%40cgiar.org%7C9bca2e95c0354359ba1808dc389bf0a5%7C6afa0e00fa1440b78a2e22a7f8c357d5%7C0%7C0%7C638447489870148251%7CUnknown%7CTWFpbGZsb3d8eyJWIjoiMC4wLjAwMDAiLCJQIjoiV2luMzIiLCJBTiI6Ik1haWwiLCJXVCI6Mn0%3D%7C0%7C%7C%7C&sdata=3FkCyT37nbsLnLfiwdZlma5eEJDuTJACD0TgISOj2zM%3D&reserved=0>. While revising your submission, please upload your figure files to the PACE digital diagnostic tool, <https://eur03.safelinks.protection.outlook.com/?url=https%3A%2F%2Fpacev2.apexcovantage.com%2F&data=05%7C02%7Csamuel.scott%40cgiar.org%7C9bca2e95c0354359ba1808dc389bf0a5%7C6afa0e00fa1440b78a2e22a7f8c357d5%7C0%7C0%7C638447489870152321%7CUnknown%7CTWFpbGZsb3d8eyJWIjoiMC4wLjAwMDAiLCJQIjoiV2luMzIiLCJBTiI6Ik1haWwiLCJXVCI6Mn0%3D%7C0%7C%7C%7C&sdata=ZVTXrOW7rVohRKX7EvKcT0sxVA4v4%2F%2B%2FsScBUmEetpo%3D&reserved=0>. PACE helps ensure that figures meet PLOS requirements. To use PACE, you must first register as a user. Then, login and navigate to the UPLOAD tab, where you will find detailed instructions on how to use the tool. If you encounter any issues or have any questions when using PACE, please email us at [PLOSMedicine@plos.org](mailto:PLOSMedicine@plos.org).

**Response:** All figures are TIF at 300 dpi and have gone through PACE, with no issues flagged.

**Comment 9:** We ask every co-author listed on the manuscript to fill in a contributing author statement, making sure to declare all competing interests. If any of the co-authors have not filled in the statement, we will remind them to do so when the paper is revised. If all statements are not completed in a timely fashion this could hold up the re-review process. If new competing interests are declared later in the revision process, this may also hold up the submission. Should there be a problem getting one of your co-authors to fill in a statement we will be in contact.  YOU MUST NOT ADD OR REMOVE AUTHORS UNLESS YOU HAVE ALERTED THE EDITOR HANDLING THE MANUSCRIPT TO THE CHANGE AND THEY SPECIFICALLY HAVE AGREED TO IT. You can see our competing interests policy here: <https://eur03.safelinks.protection.outlook.com/?url=http%3A%2F%2Fjournals.plos.org%2Fplosmedicine%2Fs%2Fcompeting-interests&data=05%7C02%7Csamuel.scott%40cgiar.org%7C9bca2e95c0354359ba1808dc389bf0a5%7C6afa0e00fa1440b78a2e22a7f8c357d5%7C0%7C0%7C638447489870156315%7CUnknown%7CTWFpbGZsb3d8eyJWIjoiMC4wLjAwMDAiLCJQIjoiV2luMzIiLCJBTiI6Ik1haWwiLCJXVCI6Mn0%3D%7C0%7C%7C%7C&sdata=YUBA6ml5g8opvFk7xVq9gdsnR2hhsbcYeGvioaS%2BSOQ%3D&reserved=0>.

**Response:** Noted. No changes made.

**Comment 10:** Please ensure that the paper adheres to the PLOS Data Availability Policy (see <https://eur03.safelinks.protection.outlook.com/?url=http%3A%2F%2Fjournals.plos.org%2Fplosmedicine%2Fs%2Fdata-availability&data=05%7C02%7Csamuel.scott%40cgiar.org%7C9bca2e95c0354359ba1808dc389bf0a5%7C6afa0e00fa1440b78a2e22a7f8c357d5%7C0%7C0%7C638447489870160624%7CUnknown%7CTWFpbGZsb3d8eyJWIjoiMC4wLjAwMDAiLCJQIjoiV2luMzIiLCJBTiI6Ik1haWwiLCJXVCI6Mn0%3D%7C0%7C%7C%7C&sdata=TCTkyU3G1ONVWRyNXzLEVfdipeu%2Fmh3w78IEquMkFPI%3D&reserved=0)>, which requires that all data underlying the study's findings be provided in a repository or as Supporting Information. For data residing with a third party, authors are required to provide instructions with contact information for obtaining the data. PLOS journals do not allow statements supported by "data not shown" or "unpublished results." For such statements, authors must provide supporting data or cite public sources that include it.

**Response:** We will make the data for the paper available.
