# Supplementary material for: Deworming and micronutrient status by community open defecation prevalence: An observational study using nationally representative data from India, 2016–2018
Source: PLoS Med. 2024 May 10;21(5):e1004402. doi: 10.1371/journal.pmed.1004402 (PMC11125536; doi:10.1371/journal.pmed.1004402)
Supplement: Attachment — Submitted filename: Deworming_OD_Response to reviewers_v2.docx [file pmed.1004402.s011.docx]

**Reviewer 1**

**Comment 1:** The authors have made extensive revisions and, upon thorough evaluation of the manuscript, it now satisfies the PLOS Medicine standards for publication.

**Response:** We thank the reviewer.

**Reviewer 2 (Michael Dewey)**

**Comment 1:** The authors have addressed my points.

**Response:** We thank the reviewer.

**Reviewer 4**

**Comment 1:** All my comments have been adequately addressed in the revised version. Thank you.

**Response:** We thank the reviewer.

**Editor**

**Comment 1:** We suggest reporting statistical information in the following format: ‘x’; (95% CI [‘y’,’ z’] p value) and use commas as opposed to hyphens (as these can be confused with negative values) to separate upper and lower bounds. For p values, please report as p<0.001 and where higher as 'p=0.002'. Please add the statistical method used to your method section. We also invite you to report p values to consistently to the third decimal digit - thousandths.

**Response:** We have made this correction.

**Comment 2:** Supplementary materials: Please note that supplementary materials are not checked and will be posted as supplied by the authors. Therefore, please double check. Please cite your Supporting Information as outlined here: <https://eur03.safelinks.protection.outlook.com/?url=https%3A%2F%2Fjournals.plos.org%2Fplosmedicine%2Fs%2Fsupporting-information&data=05%7C02%7CS.Chakrabarti%40cgiar.org%7C00c81390bd824d4a3a4708dc558f6143%7C6afa0e00fa1440b78a2e22a7f8c357d5%7C0%7C0%7C638479321500520819%7CUnknown%7CTWFpbGZsb3d8eyJWIjoiMC4wLjAwMDAiLCJQIjoiV2luMzIiLCJBTiI6Ik1haWwiLCJXVCI6Mn0%3D%7C0%7C%7C%7C&sdata=US7Vr28PWpV%2BDrFnCzgUIKnfKdJkqUd9WerfTq6nuZQ%3D&reserved=0> - Please note you may use almost any description as the item name of your supporting information as long as it contains an "S" and number. For example, “S1 Appendix” and “S2 Appendix,” “S1 Table” and “S2 Table. Please ensure each supplementary material has a call out (link) from your main manuscript.

**Response:** We have checked the supplementary material and each material has a call out from the main manuscript. We have updated the table and figure number for the supplementary material.

**Comment 3:** To help us extend the reach of your research, please provide any Twitter handle(s) that would be appropriate to tag, including your own, your coauthors’, your institution, funder, or lab.

**Response:**

Suman Chakrabarti: @SumaanCh

Sitara Ajjampur @ajjampur_s

Avinash Kishore @avinash_shahi

IFPRI: @IFPRIResearch @IFPRI @IFPRISAO

CMC Vellore @OffCMCVellore

London School @LSHTM

**Comment 4:** TITLE:

We note that your use ‘modelling study’ as the study descriptor in your title, but your study is an observational study. We suggest you change your title to: “Deworming and micronutrient status by community open defecation: An observational study using nationally representative data from India, 2016-18.", or something similar.

**Response:** We have changed the title.
DEWORMING AND MICRONUTRIENT STATUS BY COMMUNITY OPEN DEFECATION PREVALENCE: AN OBSERVATIONAL STUDY USING NATIONALLY REPRESENTATIVE DATA FROM INDIA, 2016-18

**Comment 5:** DATA AVAILABILITY:

Thank you for providing all your data on acceptance. At that time, please ensure you update your data availability statement.

**Response:** We will do this.

**Comment 6:** ABSTRACT:

Please structure your abstract using the PLOS Medicine headings: Background, Methods and Findings, Conclusions. Please note that Methods and Findings is one section. Please remove all other subheaders.

**Response:** We have made this change.

**Comment 7:** Abstract Methods and Findings:

In the opening line, please add that you mean “India” when your refer to National representation.

In the last sentence of the Abstract Methods and Findings section, please describe the main limitation(s) of the study's methodology.

**Response:** We have made this change.

**Comment 8:** AUTHORS SUMMARY:

At this stage, we ask that you include a short, non-technical Author Summary of your research to make findings accessible to a wide audience that includes both scientists and non-scientists. The Author Summary should immediately follow the Abstract in your revised manuscript. This text is subject to editorial change and should be distinct from the scientific abstract. Please see our author guidelines for more information: <https://eur03.safelinks.protection.outlook.com/?url=https%3A%2F%2Fjournals.plos.org%2Fplosmedicine%2Fs%2Frevising-your-manuscript%23loc-author-summary&data=05%7C02%7CS.Chakrabarti%40cgiar.org%7C00c81390bd824d4a3a4708dc558f6143%7C6afa0e00fa1440b78a2e22a7f8c357d5%7C0%7C0%7C638479321500530899%7CUnknown%7CTWFpbGZsb3d8eyJWIjoiMC4wLjAwMDAiLCJQIjoiV2luMzIiLCJBTiI6Ik1haWwiLCJXVCI6Mn0%3D%7C0%7C%7C%7C&sdata=OyZX9nyPb8ouSrPYl5utTe4VXiu0zjjdPF%2BGU5cFWyE%3D&reserved=0>

Ideally each sub-heading should contain 2-3 single sentence, concise bullet points containing the most salient points from your study.

**Response:** We have added the author summary.

**Comment 9:** In the final bullet point of ‘What Do These Findings Mean?’ Please include the main limitations of the study in non-technical language.

**Response:** We have added this point.
